# Supplementary material for: How Subjective Socioeconomic Status Influences Pro-Environmental Behavior: The Mediating Role of Sense of Control and Life History Strategy
Source: Behav Sci (Basel). 2024 Jul 11;14(7):591. doi: 10.3390/bs14070591 (PMC11273981; doi:10.3390/bs14070591)
Supplement: Supplementary file 1 [file behavsci-14-00591-s001.zip › behavsci-3063579-supplementary.pdf]

Supplementary Materials

How Subjective Socioeconomic Status Influences Pro-Environmental Behavior: The Mediating Role of Sense of Control and Life History Strategy

1.1 The measure of Objective SES

Objective SES includes family annual income, educational level, and occupation [14].

Specifically, **the family annual income** (including various allowances, benefits, bonuses, and rent) was measured by asking participants to choose from the following options: (1) ¥ 10,000 or below, (2) ¥ 10,001–¥ 20,000, (3) ¥ 20,001– ¥ 30,000, (4) ¥ 30,001–¥ 50,000, (5) ¥ 50,001–¥ 70,000, (6) ¥ 70,001–¥ 100,000, (7) ¥100,001–¥ 200,000, (8) ¥ 200,001–¥ 500,000, (9) ¥ 500,00–¥ 1,000,000, (10) ¥ 1,000,000 or above.

The educational level was measured by asking participants to choose from the following options: (1) elementary school or below, (2) junior high school, (3) high school/secondary vocational school/technical school, (4) junior college/night school/radio and television university, (5) undergraduate, (6) master's degree or above.

The occupation was measured based on the social stratification framework proposed by Liu (2007), which incorporates public power and market capability and adds occupational categories for the low SES and rural areas. Seven categories representing SES from low to high were obtained: (1) unemployed, semi-employed, or underemployed in urban or rural areas; (2) non-technical workers or manual laborers, such as industrial workers and agricultural laborers; (3) employees in commercial service industries, such as chefs, drivers, and hairdressers; (4) self-employed workers or business owners; (5) lower-middle class, including low-ranking officials in party and government institutions, managers of small enterprises, and junior-level professional and technical personnel; (6) upper-middle class, including middle-level leaders of party and government institutions, senior managers of large enterprises, and senior-level professional and technical personnel; and (7) upper-class, including high-level leaders of party and government institutions, senior executives of large state-owned enterprises, and private entrepreneurs.

1.2 The measurement items employing different scales

	**Constructs and Measuring Items**	*Sources*
	**Green Consumer Behavior**	
GCB1	I avoid using plastic products for environmental reasons.	
GCB2	I avoid purchasing products with excessive packaging.	
GCB3	I purchase products produced/cultivated in an environmentally friendly manner.	Study 1
GCB4	I purchase products with eco-friendly labels, indicating pollution-free products.	[41]
GCB5	I look for products packaged in reusable containers (such as glass bottles).	
GCB6	I use energy-efficient appliances.	
GCB7	I use energy-saving bulbs at home/dorm.	

	Sense of Control	
SC1	I can almost always accomplish anything I set my mind to.	
SC2	What I can and cannot do is mostly determined by others.	
SC3	If I want to do something, I can usually find a way to succeed.	
SC4	For many important things in my life, I cannot change them.	
SC5	Whether I can get what I want is within my control.	
SC6	I often feel helpless in dealing with some problems in life.	
SC7	My future mostly depends on myself.	Study 3 [24,34]
SC8	Many things that happen in my life are often beyond my control.	
SC9	When trying to do something, I am often disturbed by other things.	
SC10	I can hardly control what happens to me.	
SC11	I have no way to solve all my problems.	
SC12	Sometimes I feel like I am pushed around by others in my life.	
	Life History Strategy	
LHS1	I often can predict the outcomes of situations.	
LHS2	I think carefully about what to do before taking action.	
LHS3	I often see the positive side of unfavorable situations.	
LHS4	I don't give up before problems are solved.	
LHS5	I often make plans in advance.	
LHS6	I avoid risky behaviors.	
LHS7	During my upbringing, I maintained a close and warm relationship with my biological mother.	
LHS8	During my upbringing, I maintained a close and warm relationship with my biological father.	
LHS9	If I have or already have children, I will have a close and warm relationship with my children.	Study 3 [34,52]
LHS10	If I have or already have a partner, I will have a close and warm relationship with my significant other.	
LHS11	I remain loyal in romantic relationships.	
LHS12	Before engaging in intimate relationships with others, I establish an intimate connection with them.	
LHS13	I frequently stay in touch with my relatives.	
LHS14	I often receive emotional support and practical help from my close relatives.	
LHS15	I often provide emotional support and practical help to my close relatives.	
LHS16	I frequently maintain contact with my friends.	

LHS17	I often receive emotional support and practical help from my friends.
LHS18	I often provide emotional support and practical help to my friends.
LHS19	I frequently participate in community or group activities.
LHS20	I have a close connection to my religion or belief system.

1.3 The results of regression analysis from studies 1-3

Studies 1-3 consistently demonstrate that, even after controlling for objective SES, gender, and age, SES consistently influences pro-environmental behavior across time and different behaviors. The following are the results of the regression analysis:

Table S1. Regression analysis of subjective SES on green consumption behavior (Study 1: $N = 257$)

Variable	*b*	*SE*	*t*	*p*	95.0% CI	
					LL	UL
Intercept	5.44	0.16	33.76	< 0.001	5.12	5.76
Subjective SES	0.19	0.08	2.35	0.020	0.03	0.35
Objective SES	−0.07	0.06	−1.18	0.241	−0.18	0.05
Sex	−0.16	0.08	−1.97	0.050	−0.31	0.00
Age	0.01	0.05	2.21	0.028	0.00	0.02
R^2				0.06		
F				4.32**		

Notes. *b* = unstandardized coefficients. CI = percentile bootstrapped confidence intervals, LL = lower limit; UL = upper limit.

Table S2. Regression analysis of subjective SES on environmental donation behavior (Study 2: $N = 146$)

Variable	*b*	*SE*	*t*	*p*	95.0% CI	
					LL	UL
Intercept	7.90	1.82	4.35	<0.001	4.31	11.49
Subjective SES	3.36	0.84	3.99	<0.001	1.69	5.02
Objective SES	0.63	0.63	1.01	0.313	−0.60	1.87
Sex	0.51	0.84	0.61	0.542	−1.14	2.17
Age	0.04	0.06	0.67	0.507	−0.08	0.15
R^2				0.13		
F				5.34***		

Notes. *b* = unstandardized coefficients. CI = percentile bootstrapped confidence intervals, LL = lower limit; UL = upper limit.

Table S3. Regression analysis of subjective SES on environmental donation behavior (Study 3: $N = 351$)

Variable	*b*	*SE*	*t*	*p*	95.0% CI	
					LL	UL
Intercept	5.71	1.20	4.77	<0.001	3.36	8.07
Subjective SES	2.11	0.55	3.84	<0.001	1.03	3.19
Objective SES	0.74	0.40	1.83	0.068	−0.05	1.53
Sex	1.10	0.56	1.95	0.052	−0.01	2.20
Age	0.12	0.04	3.18	0.002	0.05	0.19
R^2				0.11		
F				10.67***		

Notes. *b* = unstandardized coefficients. CI = percentile bootstrapped confidence intervals, LL= lower limit; UL = upper limit.
